# The Associations between Self-Reported Exposure to the Chernobyl Nuclear Disaster Zone and Mental Health Disorders in Ukraine

**DOI:** 10.3389/fpsyt.2018.00032

**Published:** 2018-02-15

**Authors:** Matthew A. Bolt, Luralyn M. Helming, Nathan L. Tintle

**Affiliations:** ^1^Department of Psychology, Dordt College, Sioux Center, IA, United States; ^2^Department of Mathematics, Computer Science and Statistics, Dordt College, Sioux Center, IA, United States

**Keywords:** chernobyl nuclear accident, radiation, nuclear disaster, self-reported health status, world mental health survey, composite international diagnostic interview, Ukraine

## Abstract

**Background:**

In 1986, Reactor 4 of the Chernobyl nuclear power plant near Pripyat, Ukraine exploded, releasing highly-radioactive materials into the surrounding environment. Although the physical effects of the disaster have been well-documented, a limited amount of research has been conducted on association of the disaster with long-term, clinically-diagnosable mental health disorders. According to the diathesis–stress model, the stress of potential and unknown exposure to radioactive materials and the ensuing changes to ones life or environment due to the disaster might lead those with previous vulnerabilities to fall into a poor state of mental health. Previous studies of this disaster have found elevated symptoms of stress, substance abuse, anxiety, and depression in exposed populations, though often at a subclinical level.

**Materials and methods:**

With data from The World Mental Health Composite International Diagnostic Interview, a cross-sectional large mental health survey conducted in Ukraine by the World Health Organization, the mental health of Ukrainians was modeled with multivariable logistic regression techniques to determine if any long-term mental health disorders were association with reporting having lived in the zone affected by the Chernobyl nuclear disaster. Common classes of psychiatric disorders were examined as well as self-report ratings of physical and mental health.

**Results:**

Reporting that one lived in the Chernobyl-affected disaster zone was associated with a higher rate of alcohol disorders among men and higher rates of intermittent explosive disorders among women in a prevalence model. Subjects who lived in the disaster zone also had lower ratings of personal physical and mental health when compared to controls.

**Discussion:**

Stress resulting from disaster exposure, whether or not such exposure actually occurred or was merely feared, and ensuing changes in life circumstances is associated with increased rates of mental health disorders. Professionals assisting populations that are coping with the consequences of disaster should be aware of possible increases in psychiatric disorders as well as poorer perceptions regarding personal physical and mental health.

## Introduction

On April 26, 1984, Unit 4 of the Chernobyl Nuclear Power Plant suffered several explosions and fires, which released large amounts of radionuclides into the surrounding environment (1). Nearby populations were evacuated two days later, yet, the radioactive particles spread far beyond the initial evacuation zone, eventually contaminating an area with a population of greater than five million (1, 2). To this day, the Chernobyl nuclear disaster is considered one of the largest and most disastrous of all nuclear accidents (2, 3).

Populations affected by large-scale disasters are often prone to increased rates of mental health disorders, such as posttraumatic stress and depression, and are more likely to self-medicate through alcohol or substance abuse (4–6). Additionally, disaster-surviving populations self-report worse physical and mental health than similar non-disaster counterparts (7). This pattern is not surprising; a poor self-rating of personal health often correlates with poor psychosocial health (8). These changes in mental and physical health are theorized to be caused not only by the immediate stressor of a disaster itself but also by stress due to sudden evacuation, unexpected danger, unknown negative health effects, changes or difficulties with living situations, and familial conflict (9, 10).

What is especially unique about nuclear disasters is the ambiguity and uncertainty of the strength and extent of danger. Unlike other natural disasters like hurricanes or wildfires, where signs of damage are clear and visual or tactile, nuclear disasters have no obvious indicators of threat or danger.

Populations living nearby disaster areas lack answers to whether they are exposed and, if so, how severely. We suspect this ambiguity is critical in the development of mental health disorders in the aftermath of nuclear disasters.

With nuclear disasters, victims also bear the additional burden of stigmatization as radioactively contaminated persons (9, 11). As media coverage labels those exposed as victims, they become seen as contaminated or dirty (11). This stigma or ostracism can also lead to increased levels of social stress and can be yet another potential contributor to a higher risk of mental health problems (12).

Dramatic or significant stressors like disasters may be related to the precipitation or relapse of various mental health disorders according to the diathesis–stress model. The diathesis–stress model assumes that all individuals demonstrate various likelihoods for developing mental health disorders, whether due to genetics or environmental pressures. Under enough stress, a threshold of tolerance and one’s ability to cope can be surpassed and a mental health disorder develops (13, 14). We hypothesize that the Chernobyl nuclear disaster and ensuing stressors are adequately traumatic to be associated with increased rates of mental health disorders, especially since man-made technological disasters, specifically nuclear ones, are the most terrifying of all disasters (15, 16).

Higher rates of poor mental-health are common in populations exposed to nuclear radioactivity. Survivors of the nuclear weapons unleashed on Hiroshima and Nagasaki had higher rates of anxious and somatic symptoms decades after the bombings, especially for those living closer ground zero (17, 18).

In the Gomel region, an area of Belarus near the Chernobyl disaster, investigators used the General Health Questionnaire to measure local psychopathological symptoms. A sample from the region had much higher rates of anxious and depressive symptoms than a demographically similar group living at a greater distance from Chernobyl. However, the difference in prevalence rates of clinically diagnosable psychiatric disorders between the two groups was not statistically significant (19).

Clean-up workers (liquidators) exposed to radiation during the remediation process, or at minimum the threat of it, experienced higher rates of diagnosable mood and anxiety disorders compared to controls, yet, did not struggle significantly more with alcohol disorders nor intermittent explosive disorder [IED (20)]. Among Estonians, even 24 years after the incident, those involved in cleanup measures were three times more likely to suffer unexplained symptoms of somatization and were more likely to struggle with alcohol abuse than controls. Sleep problems were also significantly elevated as well as symptoms of agoraphobia (21).

A study involving Russian immigrants to the United States found that those who lived within 150 km of the Chernobyl disaster prior to emigration had much higher scores on the Russian Beck Anxiety Inventory and the Russian Beck Depressive Inventory than those who lived greater than 150 km from Chernobyl (22). Finally, a concluding review of several studies confirmed that populations in Belarus and Ukraine that were exposed to Chernobyl experienced greater incidences of depression, anxiety, and medically unexplained symptoms, but at levels which were not clinically diagnosable (9).

Chernobyl exposure was associated not only with poor mental health of surrounding populations, but perception of personal health was also worse. In the Russian immigrant study, those who lived closer to the site of the accident self-reported poor mental health at three times the rate of those further from the disaster (22). Another study in the Gomel region described that those who lived closer to Chernobyl self-reported poor health at much greater rates than an unaffected, distant Russian community, even though on a clinical scale no definite distinctions between the overall health of groups were observed (23).

Many of the studies undertaken thus far have been scientifically rigorous, but several shortcomings in the literature need to be addressed. Most projects have examined the mental health effects of the Chernobyl disaster effectively, but with convenience and non-representative samples; our study complements many of their objectives, but with a large, population-based representative sample of the entirety of Ukraine. Second, our survey tool allows us to make clinically based psychiatric diagnosis for various disorders based on DSM-IV qualifications. Finally, our study enables the scientific community to draw conclusions about the long-term effects of the disaster due to the 16-year gap between the accident (1986) and survey implementation (2002). Information about the long-term effects of nuclear disasters on mental health can provide insight for care workers and government agencies in future or current disaster remediation processes, like those working with the survivors of the 2011 Fukushima Daiichi nuclear disaster.

With information from the Ukraine World Mental Health Survey (Ukraine-WMH), we examined the effect of self-reporting that one had lived “in the zone contaminated as a result of the Chernobyl accident” (24, 25) on prevalence and relapse rates of psychological disorders and self-reported respondent health scores. Comparisons between groups were examined by using binomial logistic regression modeling and chi-square tests. Given highly differential rates of mental health disorders between sexes in Ukraine (26), as wells as the possibility of gender acting as a diathesis for different disorders, we looked at overall models and models stratified by sex.

## Materials and Methods

### Data Collection

Data collection took place from February to December of 2002 across all 24 of Ukraine’s oblasts (states) and the republic of Crimea using the Ukraine version of the World Mental Health- Composite International Diagnostic Interview (CIDI), led by the World Health Organization-World Mental Health cross-national research initiative (24–26). The survey had a 78.3% response rate, for a total sample of 4,725 respondents, and used a cross-sectional, four-tier, multi-stage, cluster sampling design (26). Survey questions provided adequate information to make WHO approved DSM-IV diagnoses for a variety of mental health disorders (26). The sample was equipped with weights based on demographic information to accurately represent the full adult Ukrainian population (26). The survey instrument was translated from English into Russian and Ukrainian with WHO-endorsed methods (26).

### Measures

We analyzed four classes of disorders found in the Ukraine WMH-CIDI: alcohol disorders (with or without dependence), affective disorders (major depression and dysthymia), anxiety disorders (social phobia, agoraphobia, generalized anxiety disorder and panic disorder), and intermittent explosive disorder (IED). IED was included in analysis as a disorder involving impulse control, and is typically included in other analyses of this sample. Unfortunately, due to sample size restrictions and the two-tier survey design, we were unable to include measures on posttraumatic stress disorder (PTSD) in the analysis as well as any common sleep disorders.

The CIDI yields information about one’s mental health status at the time of data collection, as well as if the respondent had a distinct and separate disorder that met the full DSM-IV criteria previous to data collection. We used current mental health status as our main dependent variable and adjusted for this outcome with participants’ history or lack of mental health illness, as well as other pertinent demographic variables. The answer to the question “In general, would you say your (physical and mental) health is excellent, very good, good, fair, or poor?” formed our metric for self-reported health (24–26).

Respondents also answered the question, “Have you ever lived in the zone contaminated as a result of the Chernobyl accident?” (24–26). Responses to this question (yes = Chernobyl sample; no = non-Chernobyl sample) formed our explanatory variable of interest in this study. This question was asked near the very end of the survey; therefore, respondents were not primed to recall their mental health history in light of potential exposure to Chernobyl.

Demographic information collected included sex, age, level of education, marital status, employment, financial resources, and region of the country where respondents lived at the time of data collection.

### Data Analysis

Data was analyzed with R 3.4.0, using the “survey” package to account for the complex survey design and weighting schemes (27). We used proportions, counts, means, and chi*-*squared or *t*-tests to summarize demographic characteristics of the Chernobyl and non-Chernobyl samples and to demonstrate demographic differences between the two groups.

To test for potential associations of living in the affected zone with mental health, we used logistic regression modeling to predict mental health disorder prevalence by exposure status, fitting five separate models—one for each class of DSM-IV mental health disorders, as well as one for the presence of any disorders of our four classes. We also created these models for men and women separately, examining differences in prevalence rates of disorders per sex. We suspected that gender could potentially be a moderating vulnerability factor for different disorders according to the diathesis–stress model.

Relapse rates of those who had mental health disorders before the disaster were also analyzed in separate models.

Finally, we examined self-reported health by Chernobyl exposure status with a chi-squared test, then distributed respondents into two groups to predict self-reported health with logistic regression modeling. We additionally utilized non-logistic linear regression modeling analyze this variable, treating responses as ordinal.

All models were first fit using only the Chernobyl exposure variable; then, full adjusted models were fit using the Chernobyl exposure variable as well as seven pertinent demographic variables (sex, age, education, employment, marital status, financial resources, and region of Ukraine). For the prevalence models, we also adjusted for previous mental health disorders. A significance level of 0.05 was used for all tests.

## Results

### Sample Characteristics

In the weighted sample, 8.2% (388/4,725) of respondents answered that they had lived in the Chernobyl-contaminated zone. Of those who had lived in the zone, significantly more were men, married, employed, and had attended university (Table 1). Mean and variance for age was uniform between groups. Among all respondents, a large majority had inadequate (51.1%) or very inadequate (30.8%) financial resources. Respondents were categorized as having very inadequate financial resources if they did not have enough money to buy food, and categorized as having inadequate resources if it was difficult for him or her to buy clothing and shoes. All others were categorized as having adequate financial resources. As expected, approximately two-thirds of those who said they had at lived in the zone contaminated by Chernobyl currently lived in the North-Central region of Ukraine.

**Table 1:** Sample demographics.

		Overall sample (*n* = 4,725)	Chernobyl (*n* = 388)	Non-Chernobyl (*n* = 4,336)	*F* or *t*-statistic
Chernobyl	% (*n*)	8.2 (388)			
Sex, male	% (*n*)	45.0 (2,125)	53.7 (208)	44.2 (1,916)	17.35***
Age	m (SD)	46.1 (17.7)	45.6 (15.8)	46.2 (17.9)	-0.62
Attended university	% (*n*)	44.09 (2,083)	53.8 (209)	43.2 (1,874)	6.43*
Married, yes	% (*n*)	59.8 (2,825)	65.5 (254)	59.3 (2,569)	5.72*
Employed, yes	% (*n*)	50.0 (2,361)	57.3 (222)	49.4 (2,139)	9.78**
Financial resources	% (*n*)				
Very inadequate		30.8 (1,436)	29.2 (113)	30.9 (1,323)	1.78
Inadequate		51.1 (2,348)	48.7 (188)	51.3 (2,196)	
Adequate		18.2 (848)	22.1 (85)	17.8 (763)	
Region	% (*n*)				60.20***
North-central		33.3 (1,574)	66.1 (256)	30.4 (1,316)	
West		24.7 (1,168)	12.6 (49)	25.8 (1,120)	
Southeast		42.0 (1,983)	21.3 (83)	43.8 (1,900)	

***p < 0.001; **p < 0.01; *p < 0.05.

### Mental Health Prevalence after Chernobyl

Table 2 summarizes the differences in classes of mental health disorders between those who lived in the Chernobyl zone and those who did not live in the Chernobyl zone. Prevalence rates of alcohol use disorders and IED were both significantly higher in the Chernobyl sample (18.8–11.3% for alcohol use; 6.0–4.0% for IED). These differences remained significant even after adjustment [alcohol disorders: aOR = 1.69; 95% confidence interval (CI) 1.05, 2.71; *p* = 0.040; IED: aOR = 1.56; 95% CI 1.07, 2.28; *p* = 0.0282]. Post-1986 prevalence rates of anxiety and affective disorders were not significantly different before or after adjustment, though rates of affective disorder were higher in the Chernobyl sample. When investigating rates of any mental health diagnosis post-Chernobyl, rates were higher in the Chernobyl sample than the non-Chernobyl sample, though this result was no longer statistically significant after adjusting for demographic variables and the presence of a previous mental health disorder.

**Table 2:** Mental health disorder prevalence after Chernobyl

Disorder	Chernobyl	% (*n*)	OR (95% CI)	aOR^a^ (95% CI)
Alcohol	Yes	18.8 (73)	1.82 (1.31, 2.52)***	1.69 (1.05, 2.71)*
	No	11.3 (491)	1	1
Anxiety	Yes	6.1 (24)	0.91 (0.60, 1.40)	0.71 (0.48, 1.07)
	No	6.7 (289)	1	1
Affective	Yes	18.0 (70)	1.30 (0.92, 1.84)	1.29 (0.87, 1.91)
	No	14.4 (625)	1	1
Intermittent explosive disorder	Yes	6.0 (23)	1.52 (1.08, 2.15)*	1.56 (1.07, 2.28)*
	No	4.0 (173)	1	1
Any	Yes	36.8 (143)	1.48 (1.07, 2.04)*	1.35 (0.92, 1.98)
	No	28.3 (1,227)	1	1

*OR, odds ratio; aOR, adjusted odds ratio; CI, confidence interval*.

*^a^Adjusted for sex, age, education, marital status, employment, financial resources, region, and the presence of a previous mental health disorder*.

***p < 0.001; *p < 0.05.

When stratified by sex, men in the Chernobyl zone had significantly higher rates of alcohol disorders (aOR = 1.84; 95% CI 1.09, 3.09; *p* = 0.0294). Affective disorders were also significantly higher in the model with exposure as a single predictor, but lost significance with adjustments. Rates of IED were similar between groups (Table 3). Conversely, women had significantly higher rates of IED (aOR = 2.70; 95% CI 1.52, 4.80; *p* = 0.002), but alcohol disorder rates did not vary between groups (aOR = 0.58; 95% CI 0.19, 1.83; *p* = 0.364; Table 4).

**Table 3:** Male prevalence model.

Disorder	Chernobyl	% (*n*)	OR (95% CI)	aOR^a^ (95% CI)
Alcohol	Yes	33.8 (70/208)	1.79 (1.19, 2.71)**	1.84 (1.09, 3.09)*
	No	22.2 (425/1,916)	1	1
Anxiety	Yes	3.4 (7/208)	0.79 (0.35, 1.81)	0.49 (0.18, 1.35)
	No	4.3 (82/1,916)	1	1
Affective	Yes	13.4 (28/208)	1.68 (1.02, 2.76)*	1.43 (0.77, 2.66)
	No	8.5 (162/1,916)	1	1
Intermittent explosive disorder	Yes	5.6 (12/208)	1.24 (0.66, 2.30)	0.84 (0.43, 1.64)
	No	4.6 (89/1,916)	1	1
Any	Yes	44.0 (92/208)	1.76 (1.17, 2.63)**	1.61 (1.00, 2.58)
	No	30.9 (592/1,916)	1	1

*OR, odds ratio; aOR, adjusted odds ratio; CI, confidence interval*.

*^a^Adjusted for sex, age, education, marital status, employment, financial resources, region, and the presence of a previous mental health disorder*.

**p < 0.01; *p < 0.05.

**Table 4:** Female prevalence model.

Disorder	Chernobyl	% (*n*)	OR (95% CI)	aOR^a^ (95% CI)
Alcohol	Yes	1.5 (3/180)	0.55 (0.17, 1.77)	0.58 (0.19, 1.83)
	No	2.7 (66/2,420)	1	1
Anxiety	Yes	9.3 (17/180)	1.09 (0.64, 1.88)	0.96 (0.63, 1.46)
	No	8.6 (207/2,420)	1	1
Affective	Yes	23.2 (42/180)	1.28 (0.86, 1.91)	1.32 (0.81, 2.17)
	No	19.1 (463/2,420)	1	1
Intermittent explosive disorder	Yes	6.3 (11/180)	1.86 (1.16, 2.99)**	2.70 (1.52, 4.80)***
	No	3.5 (85/2,420)	1	1
Any	Yes	28.4 (51/180)	1.12 (0.75, 1.66)	1.12 (0.76, 1.67)
	No	26.2 (634/2,420)	1	1

*OR, odds ratio; aOR, adjusted odds ratio; CI, confidence interval*.

*^a^Adjusted for sex, age, education, marital status, employment, financial resources, region, and the presence of a previous mental health disorder*.

***p < 0.001; **p < 0.01.

### Mental Health Disorder Relapse

We also examined disorder relapse; that is, if respondents who experienced mental health disorders before the disaster were more likely to have recurring problems after the accident. Those who had any mental health disorders before the disaster and reported that they had lived in the zone affected by the disaster relapsed into another episode of a mental health disorder at a rate of 21.9%, while those who did not report they had been affected by the disaster relapsed at a rate of 14.7% (aOR = 1.58; 85% CI 1.09, 2.27; *p* = 0.021).

### Self-Reported Health

Chernobyl also played a significant role in respondents’ perception of personal health [chi-square *p* = 0.04 (Table 5)]. More individuals rated their health as fair (49.8 vs 44.5%) or poor (25.4 vs. 21.1%) in the Chernobyl group compared to the non-Chernobyl group. When comparing self-reported health on a dichotomous scale, the proportion of the Chernobyl sample that rated personal health as fair or poor was significantly larger than the non-Chernobyl group (75.2 vs. 65.6%; OR = 1.6; 95% CI 1.13, 2.25; *p* = 0.012), a result that remained significant after adjustment (aOR = 1.87; 95% CI 1.28, 2.74; *p* = 0.003). Results were also significant when health ratings were treated as an ordinal variable (rating 1–5) in both unadjusted [beta = 0.16, SE(beta) = 0.06; *p* = 0.006] and adjusted models [beta = 0.19, SE(beta) = 0.05; *p* = 0.002].

**Table 5:** Distribution of rate-health responses.

Rating	Chernobyl sample% (*n*)	Non-Chernobyl sample% (*n*)
Excellent (1)	1.7 (7)	2.4 (103)
Very good (2)	1.9 (7)	3.2 (141)
Good (3)	21.2 (82)	28.8 (1,249)
Fair (4)	49.8 (193)	44.5 (1,926)
Poor (5)	25.4 (98)	21.1 (912)

## Discussion

Our analyses showed that some rates of clinically diagnosable DSM-IV mental health disorders are more prevalent in Ukrainians who reported that they lived in the zone affected by the Chernobyl disaster than Ukrainians who did not report that they were affected by the disaster. Specifically, prevalence of alcohol use disorders and IED was higher for those who lived in the zone affected by Chernobyl when compared to non-affected controls. By gender, men were at a greater risk of having alcohol disorders, while women experienced an increased risk of IED. Finally, individuals who reported living in the Chernobyl-affected zone rated their overall physical and mental health significantly worse than those who lived elsewhere in Ukraine. Under the theoretical framework of the diathesis–stress model, we conclude that the threat of exposure to radionuclides, whether real or imagined, may have been adequately stressful for those with prior diatheses to develop psychiatric disorders, although due to the nature of disaster research and our dataset, we are unable to make causal statements about this robust association.

Our results agree with the findings of previous studies, which conclude that rates of mental health disorders are likely to rise in populations affected by disaster (3–6, 8), and specific studies that report higher levels of distress and mental anguish from the Chernobyl disaster and others like it (7, 9–11, 17–23, 28, 29). Our findings regarding increased rates of alcohol use disorders and IED, and our lack of stimulating findings for anxiety disorders are interesting, especially considering previous research, which suggests anxiety disorders may be the most susceptible class of disorders to large-scale traumatic events (4, 9, 21–23, 29–33). Perhaps one reason anxiety disorders were not identified at a significantly higher rate among those reporting Chernobyl exposure compared to controls is due to our failure to include PTSD in our analyses. Unfortunately, PTSD was not assessed on all participants who took the Ukraine WHM-CIDI; the resulting small sample size hindered its inclusion in the anxiety disorders analysis.

Furthermore, we found that self-reported health ratings were significantly lower for the population exposed to Chernobyl. This finding confirms previous conclusions about self-reported health in other of disaster-surviving populations (8), as well as other studies focused specifically on the Chernobyl disaster (7, 22, 23).

Although inclusive for a wide range of health-related concerns, our study focused only on psychiatric disorders and mental health, rather than physical outcomes from radionucleotide exposure. The physical effects of the disaster and subsequent radioactive contamination have been well-documented elsewhere (1, 34).

One interesting aspect of our investigation was the time elapsed from disaster to data collection. Data collection took place in 2002, 16 years after the disaster. With such a large window between the disaster and data collection, the presence of disorders due to Chernobyl or the memory of them might have faded. Yet, this elapsed time was also an advantage, giving us the opportunity to observe the long-term effects of the disaster beyond several months or years.

A limitation of our analyses is that the false negative (respondents who said they did not live in the contaminated zone but did) and false positive (respondents who reported living in the contaminated zone but did not) rates are unknown. The survey instrument also did not inquire about severity of exposure. Furthermore, our cross-sectional design limits our understanding of the process or timeframe through which subjects developed mental health disorders or coped. Even with these limitations, however, we found significant differences in rates of mental health disorders associated with perceived exposure to the Chernobyl disaster.

Our findings confirm the conclusions of other related studies, but add scientific rigor to the conversation surrounding the effects of Chernobyl by using a population-based representative sample of Ukraine, a survey instrument with WHO-approved DSM-IV diagnostic capabilities, and a long-term timescale.

Given ours and others’ results concerning the Chernobyl disaster (1, 7, 9, 19–23) and research on the effects of nuclear weapons on public mental health (17, 18), current patterns that researchers have observed recently in Fukushima survivors (35) might continue for another decade, if not more. Our results may prove helpful in predicting the long-term outcomes of other nuclear disasters. If so, we, like Kunii et al. (35), encourage ongoing concern and monitoring for those affected by the Fukushima nuclear disaster. Further discussion and understanding of the ambiguous threats of nuclear disasters on public mental health can educate care workers and government agencies in preparation for future nuclear disasters, and guide continued care for those still suffering from these tragedies.

## Ethics Statement

This study was carried out in accordance with the recommendations of the Committees on Research Involving Human Subjects of Stony Brook University as well as the Kiev International Institute of Sociology (KIIS) and the Ukrainian Psychiatric Association (UPA) internal review boards, with written informed consent from all subjects. All subjects gave written informed consent in accordance with the Declaration of Helsinki. The protocol was approved by the Committees on Research Involving Human Subjects of Stony Brook University as well as the KIIS and the UPA internal review boards.

## Author Contributions

NT, LH, and MB developed the research plan, and MB performed statistical analysis and drafted initial versions of the manuscript. All authors approved the final version of the manuscript.

## Conflict of Interest Statement

This research was conducted in the absence of any commercial or financial relationships that could be construed as a potential conflict of interest.
